# Supplementary material for: Achievement of the Selectivity of Cytotoxic Agents against Cancer Cells by Creation of Combined Formulation with Terpenoid Adjuvants as Prospects to Overcome Multidrug Resistance
Source: Int J Mol Sci. 2023 Apr 28;24(9):8023. doi: 10.3390/ijms24098023 (PMC10178335; doi:10.3390/ijms24098023)

Figure S1. Confocal laser scanning images of A549 after 45 min incubation with Dox 5 μg/mL: (a) free, (b) with EG, (c) with apiol, (d) with preincubated EG for 30 min, (e) with preincubated apiol for 30 min. The scale segment is 30 μm (division value is 6 μm); 2 channels are shown: red, Dox; and overlay transmission light mode with Dox channel. λ_{em} = 488 nm (multiline Argon laser).

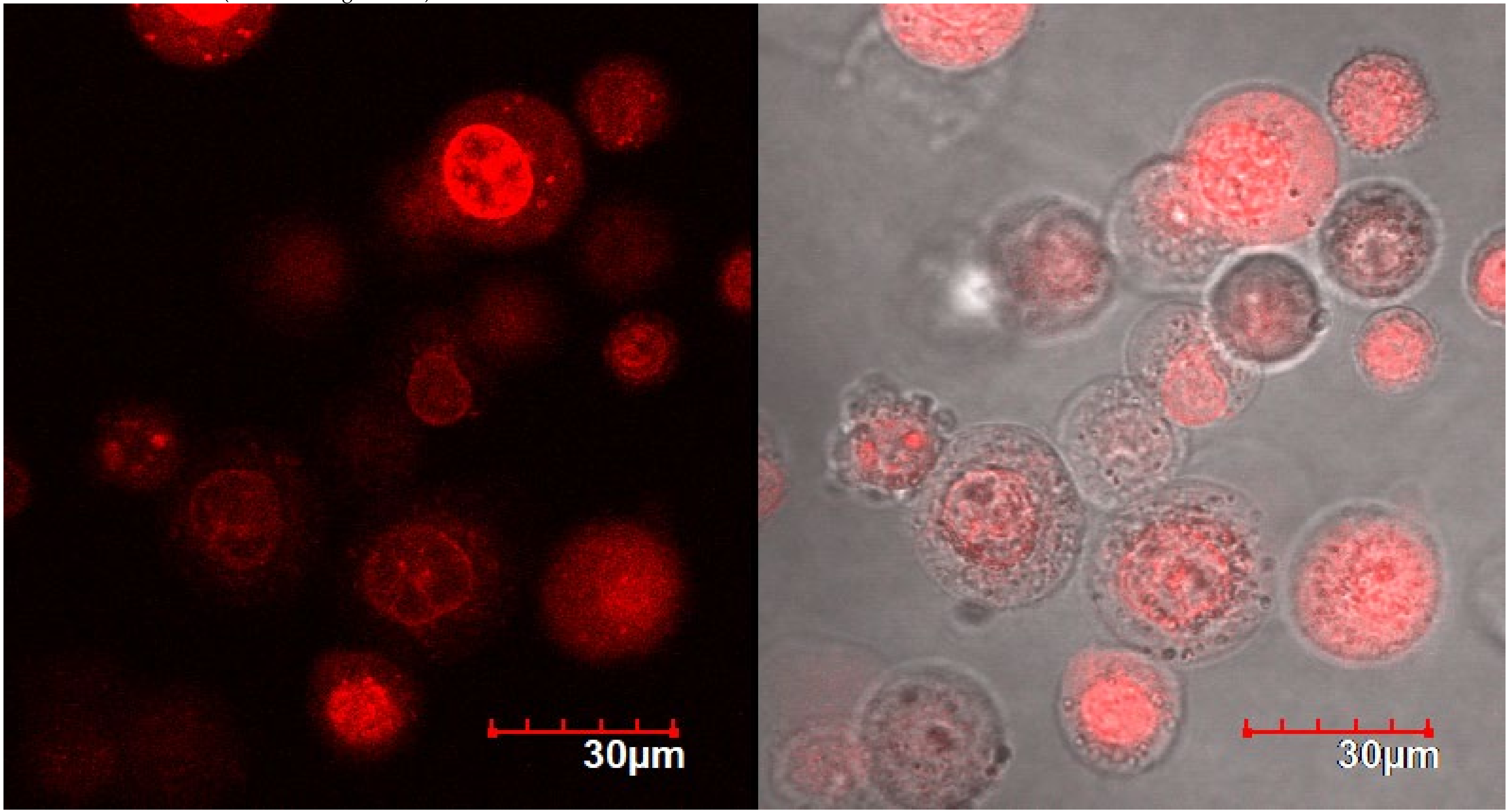

(a)

(b)

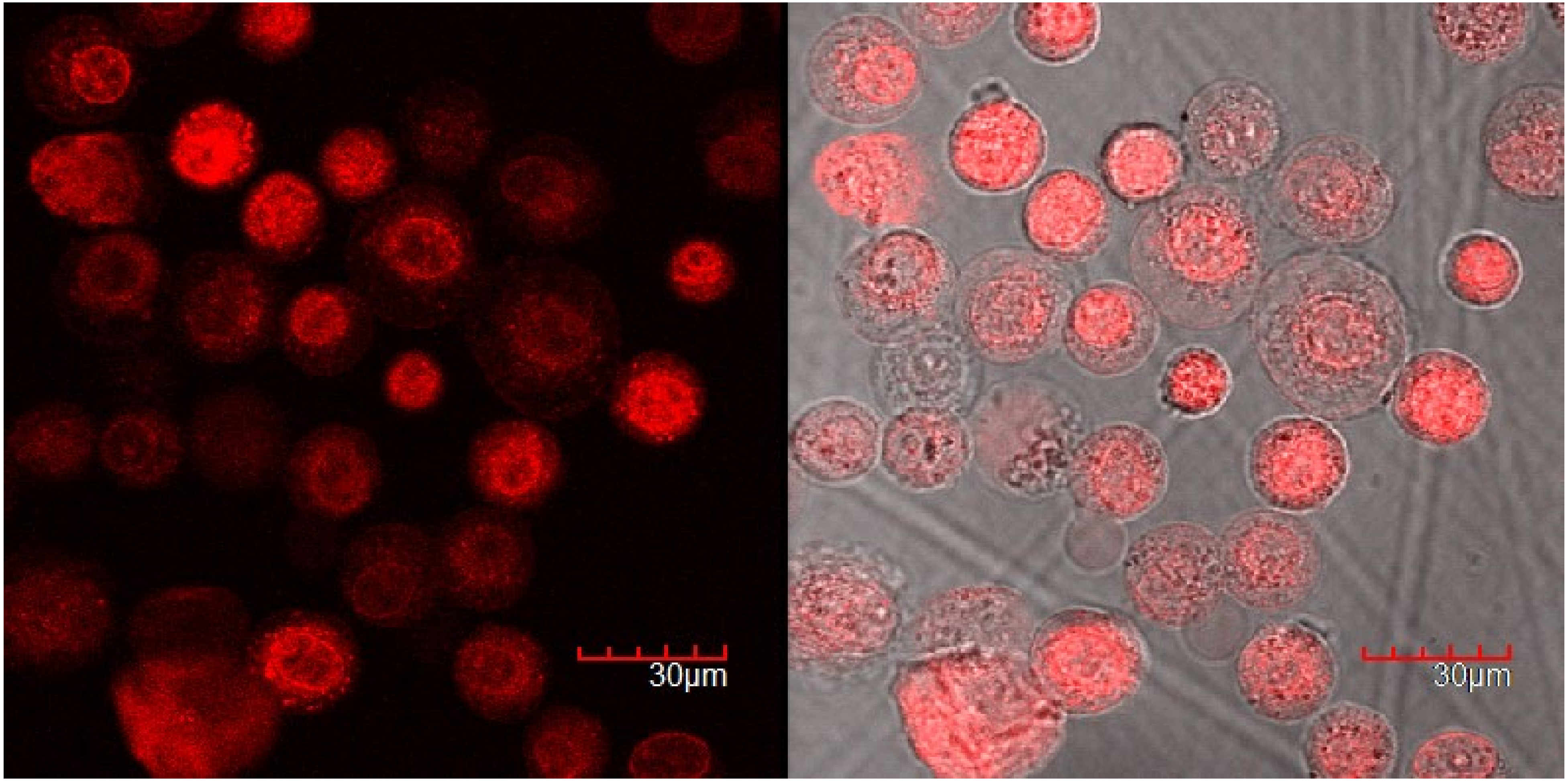

(c) 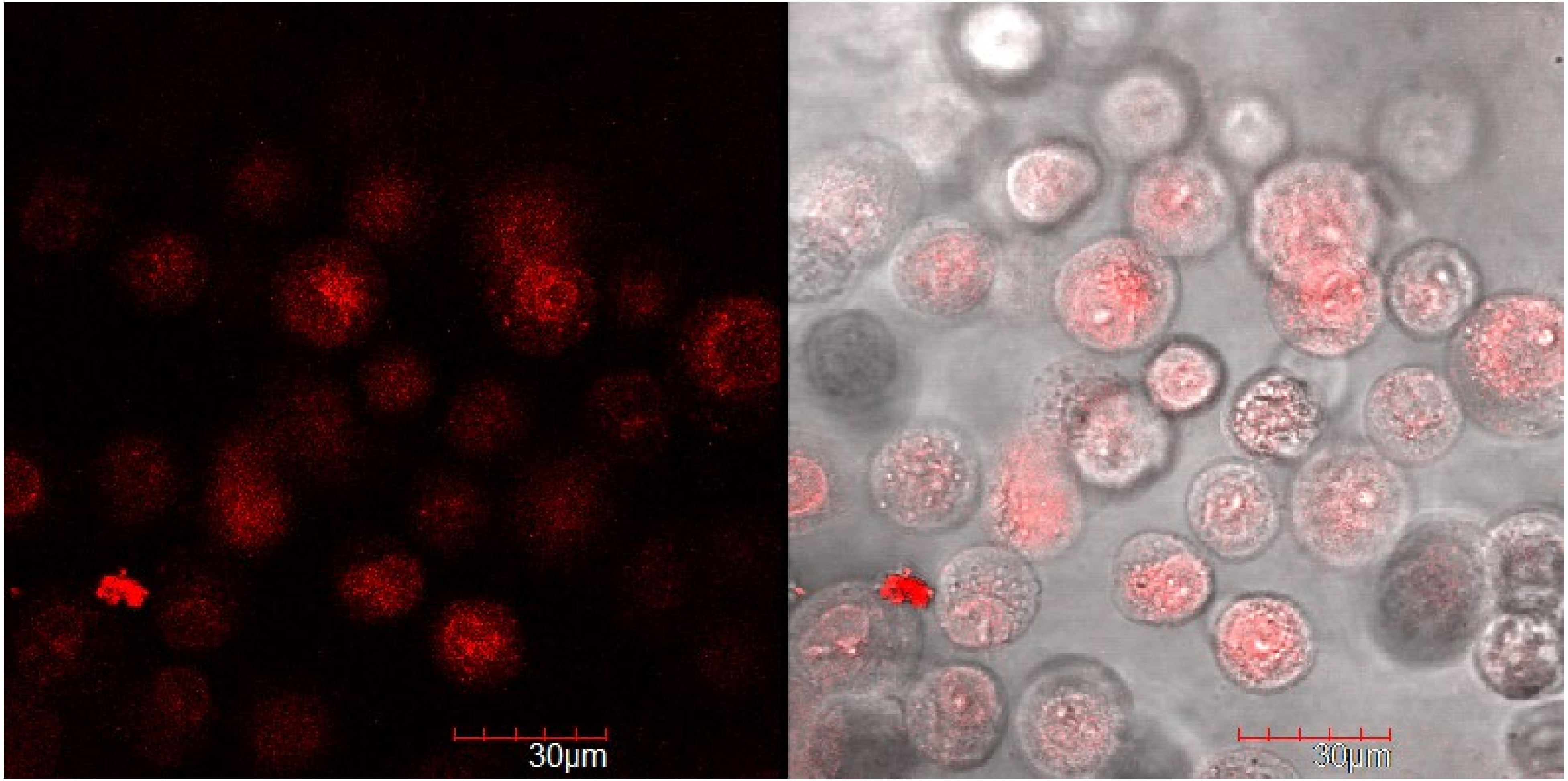

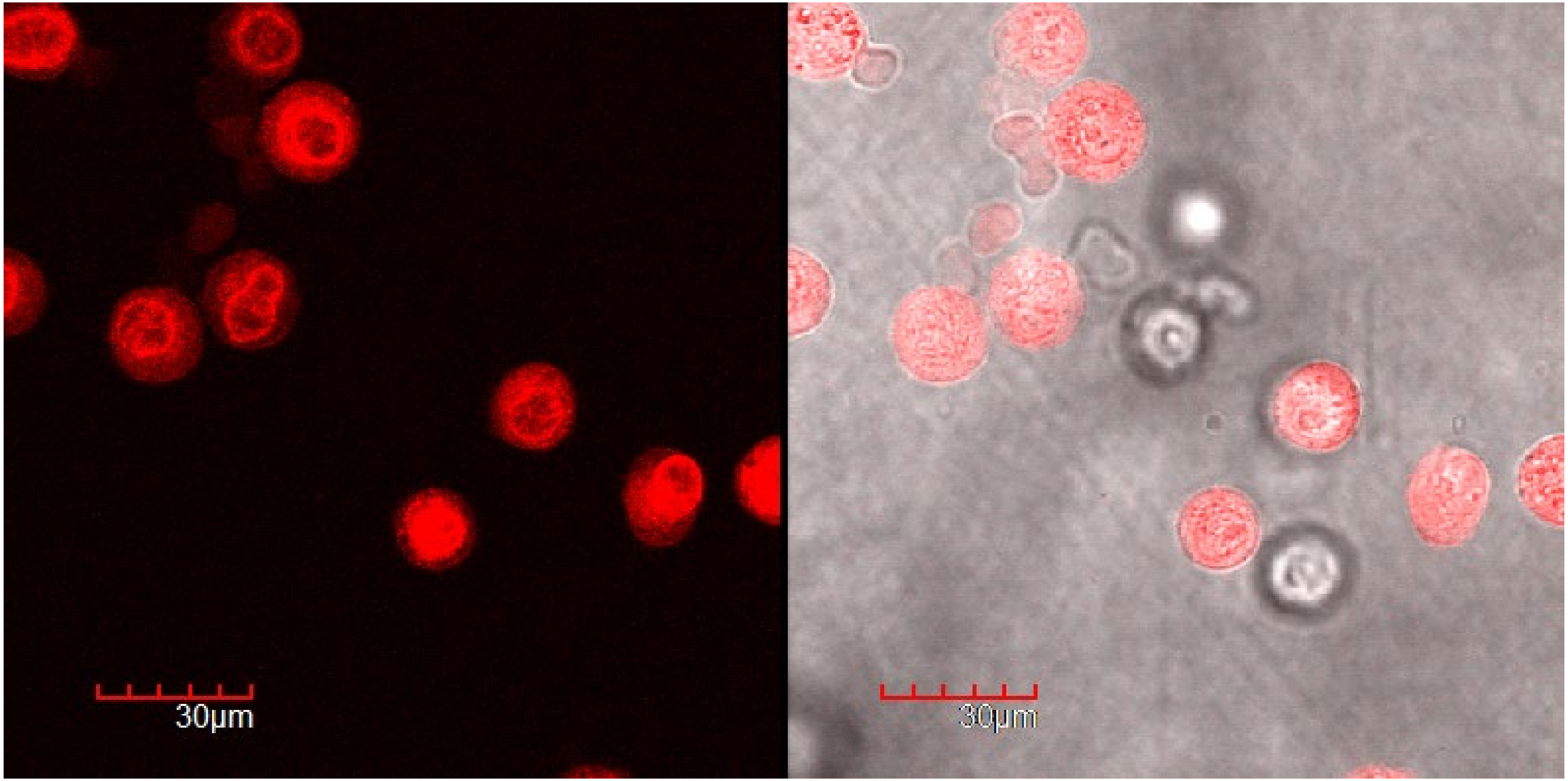

(d)

(e)

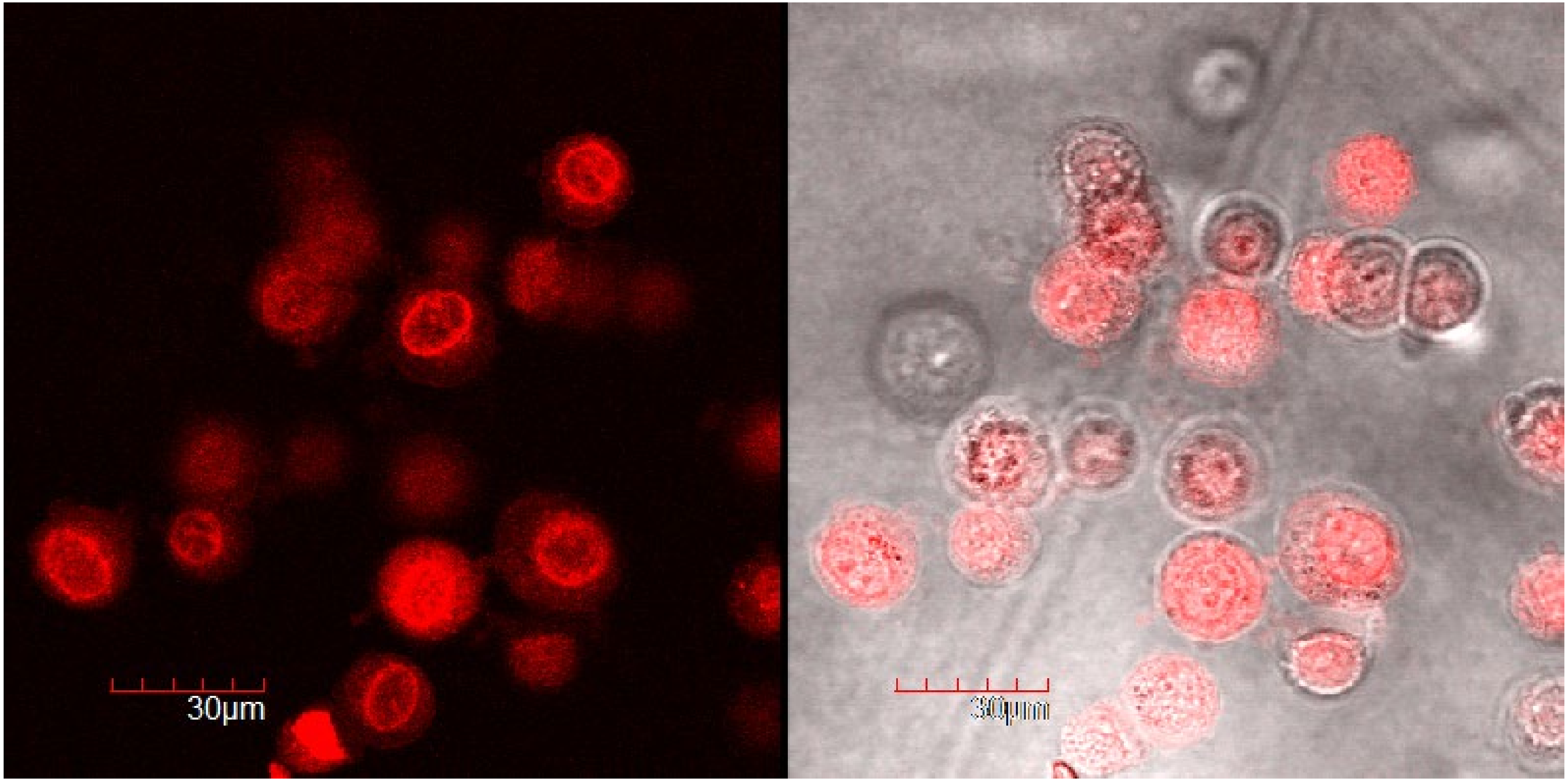

Figure S2. Confocal laser scanning images of A549 after 120 min incubation with Dox 5 μg/mL: (a) free, (b) with EG, (c) with apiol, (d) with preincubated EG for 30 min, (e) with preincubated apiol for 30 min. The scale segment is 30 μm (division value is 6 μm); 2 channels are shown: red, Dox; and overlay transmission light mode with Dox channel. λ_{em} = 488 nm (multiline Argon laser).

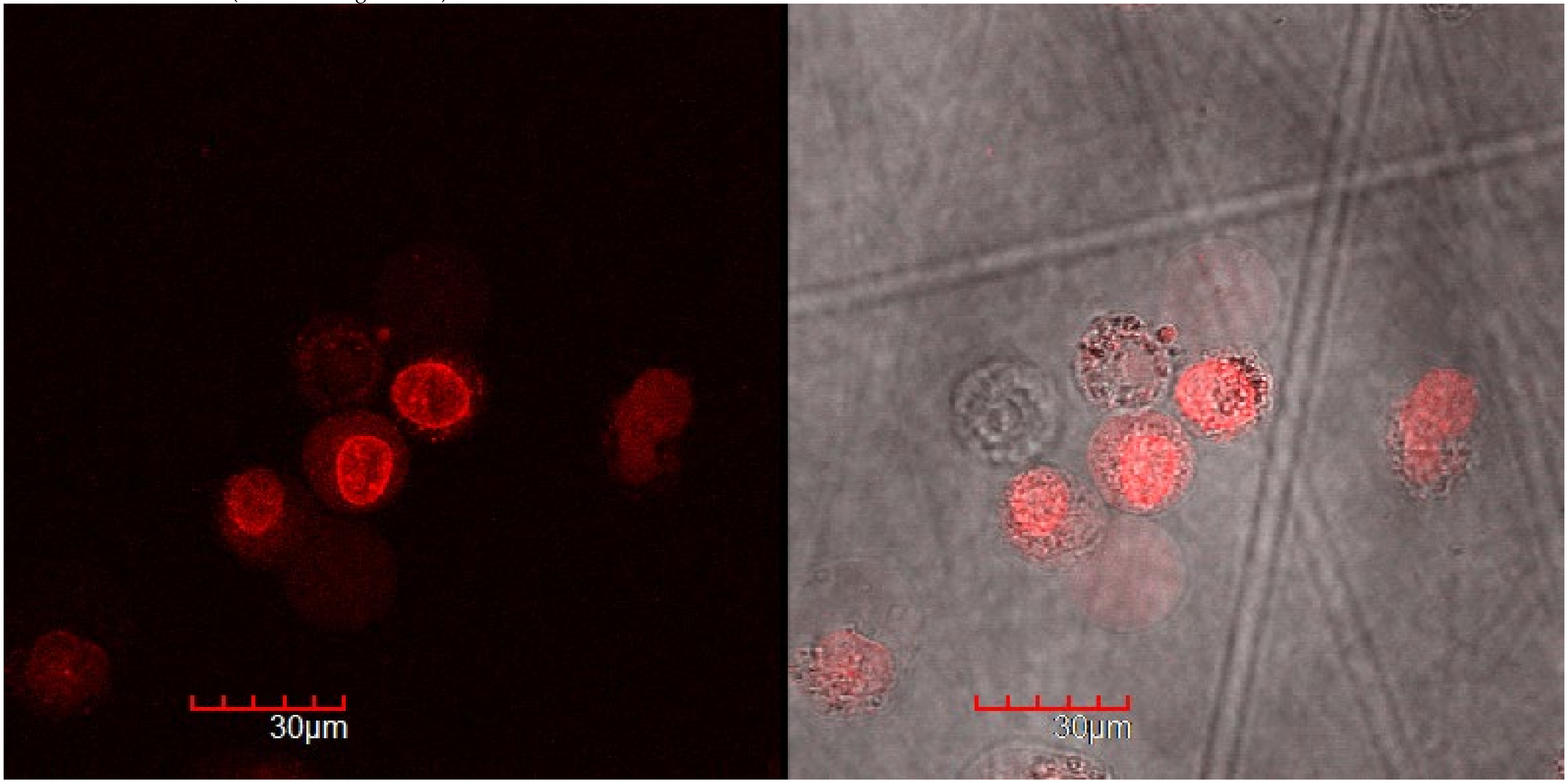

(a)

(b)

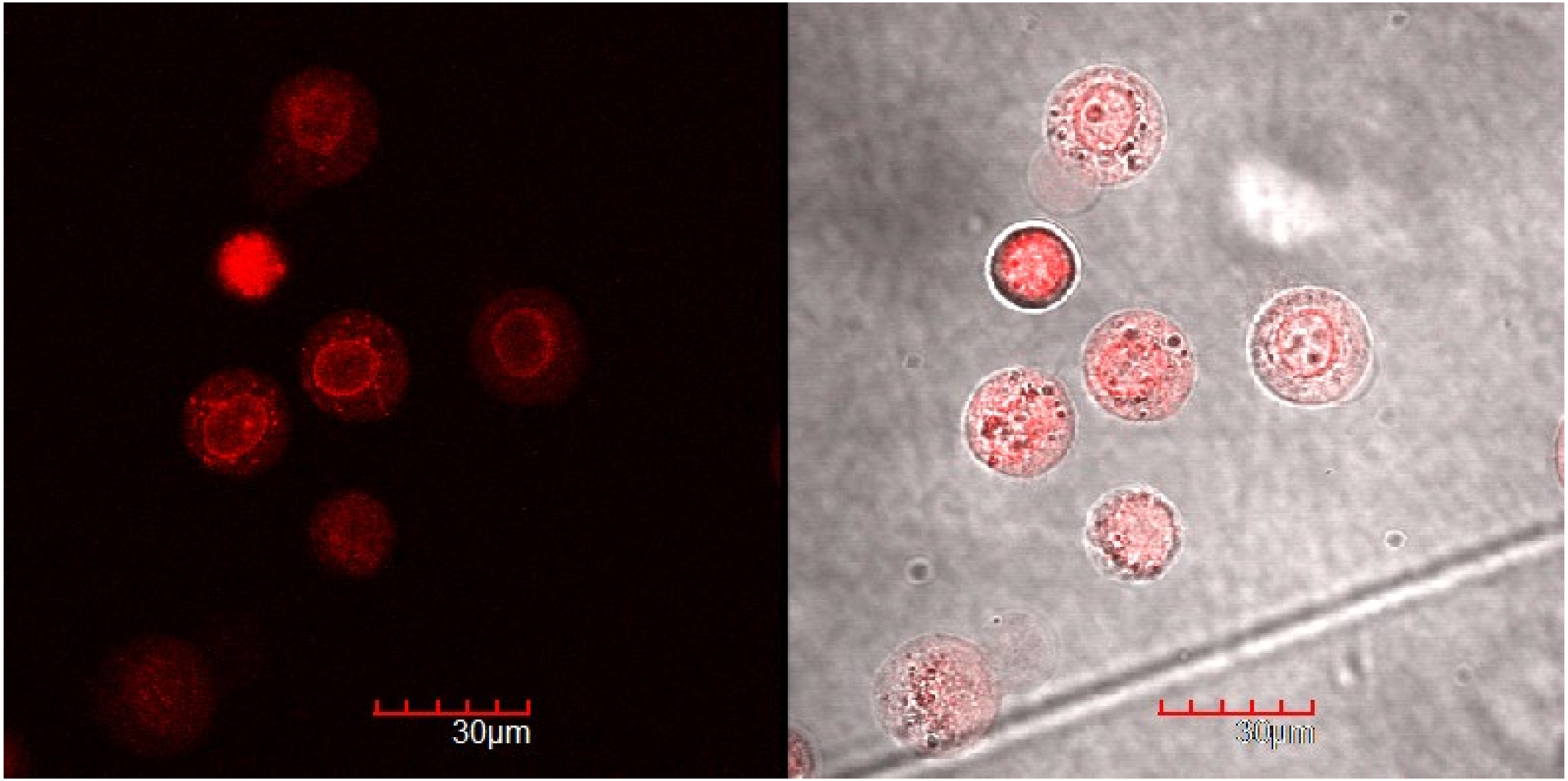

(c)

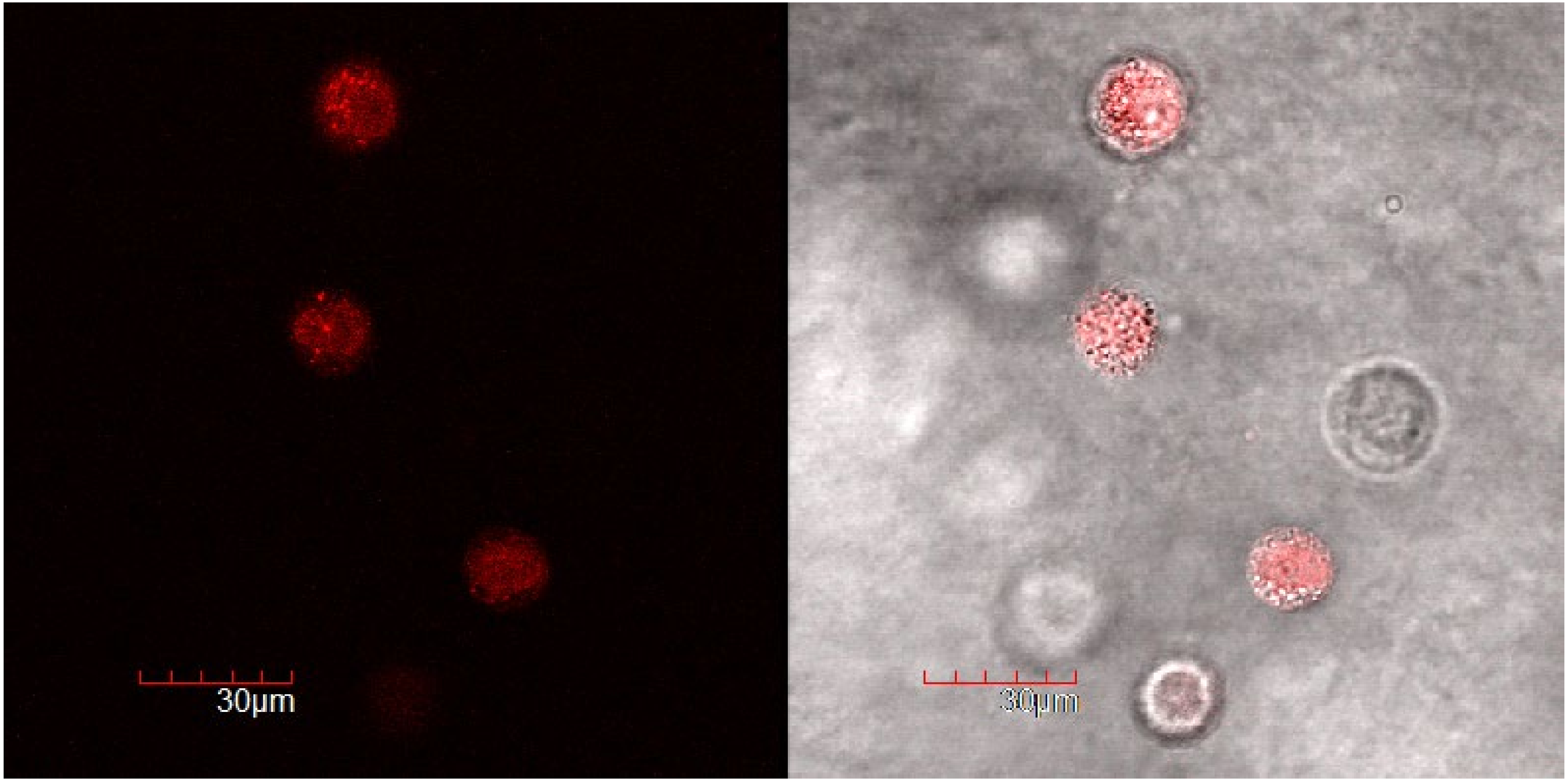

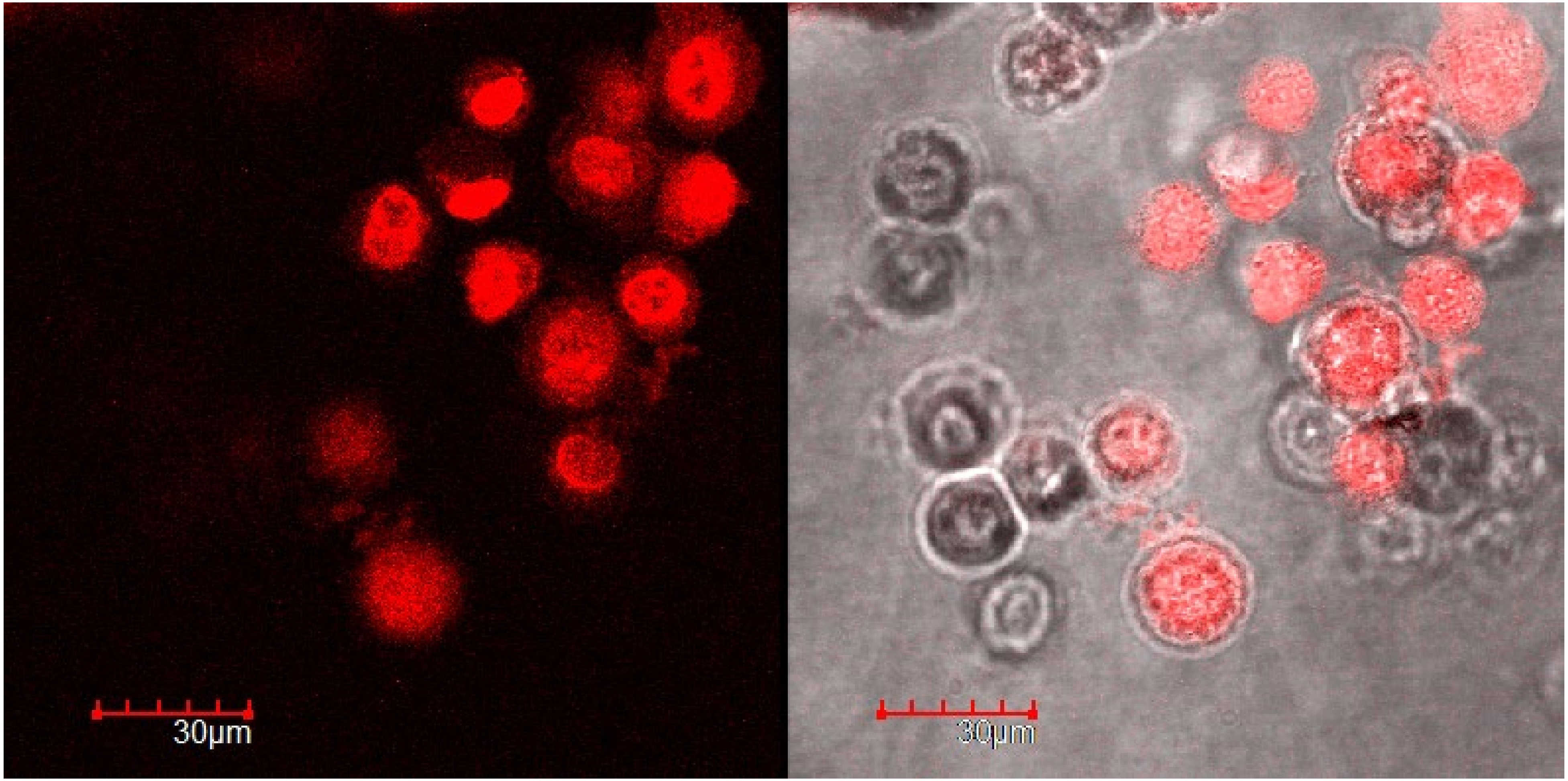

(d)

(e) 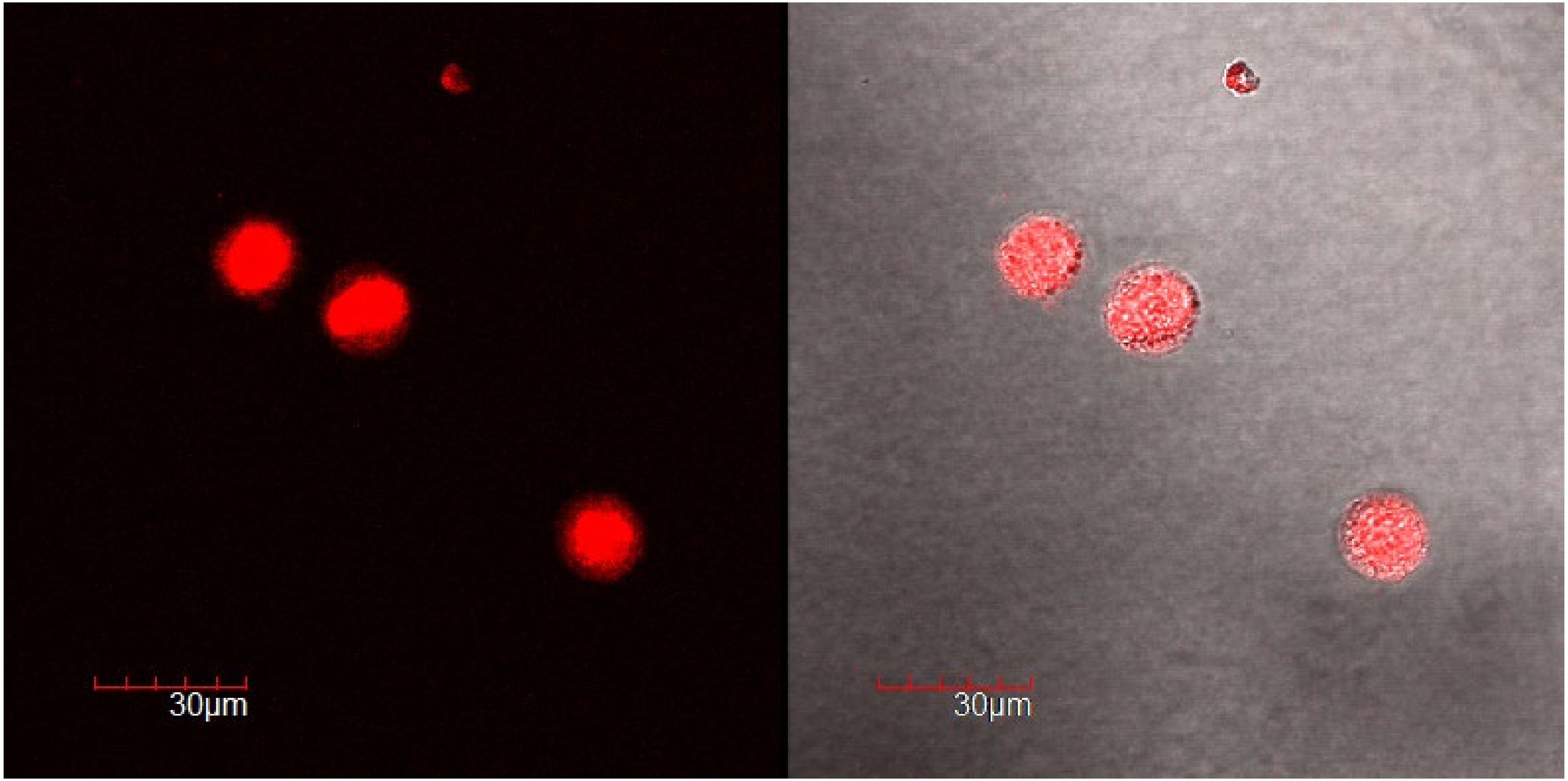

Supplement: Supplementary file 1 [file ijms-24-08023-s001.zip › ijms-2297011-supplementary.pdf]
